# Disrupted breath, songlines of breathlessness: an interdisciplinary response

**DOI:** 10.1136/medhum-2018-011631

**Published:** 2019-08-01

**Authors:** Alice Malpass, James Dodd, Gene Feder, Jane Macnaughton, Arthur Rose, Oriana Walker, Tina Williams, Havi Carel

**Affiliations:** 1 Centre for Academic Primary Care, Population Health Sciences, Bristol Medical School, University of Bristol, Bristol, UK; 2 Academic Respiratory Unit, University of Bristol, Southmead Hospital, Bristol, UK; 3 Centre for Medical Humanities, School of Medicine, Durham University, Durham, UK; 4 Department of English, University of Bristol, Bristol, UK; 5 Berlin Center for the History of Knowledge and Humboldt University, Philosophische Fakultät, Institut für Geschichtswissenschaften, Humboldt-Universität zu Berlin, Berlin, Germany; 6 Department of Philosophy, University of Bristol, Bristol, UK

**Keywords:** sciencehumanities, breathlessness, inter-disciplinary

## Abstract

Health research is often bounded by disciplinary expertise. While cross-disciplinary collaborations are often forged, the analysis of data which draws on more than one discipline at the same time is underexplored. Life of Breath, a 5-year project funded by the Wellcome Trust to understand the clinical, historical and cultural phenomenology of the breath and breathlessness, brings together an interdisciplinary team, including medical humanities scholars, respiratory clinicians, medical anthropologists, medical historians, cultural theorists, artists and philosophers. While individual members of the Life of Breath team come together to share ongoing work, collaborate and learn from each other’s approach, we also had the ambition to explore the feasibility of integrating our approaches in a shared response to the same piece of textual data. In this article, we present our pluralistic, interdisciplinary analysis of an excerpt from a single cognitive interview transcript with a patient with chronic obstructive pulmonary disease. We discuss the variation in the responses and interpretations of the data, why research into breathlessness may particularly benefit from an interdisciplinary approach, and the wider implications of the findings for interdisciplinary research within health and medicine.

## Background

If we wear theories in our boots,1 it is our disciplinary training which provides us with a compass and binoculars. It is our disciplinary trainings we rely on to orient us to the research question and identify a direction to travel as we traverse the landscape of knowledge-making. Sometimes we may become curious to see what happens if we put the disciplinary apparatus down and walk unhindered into other terrains, perhaps even stumble on common (disciplinary) ground; at different times our safe arrival (career progression, grant success) depends on our disciplinary orientation. In this article, we were interested in what happens to disciplinary identities when we work across disciplines and invite “different vantage points [from] which to view a topic”.2

## The Life of Breath project

The Life of Breath project brings together researchers from the following disciplines: medical humanities, philosophy, anthropology, history of science, English, respiratory medicine, general practice, dance and drama, arts and health, as well as collaborators who are composers, visual artists, writers, poets and music therapists. The project collaborates with the British Lung Foundation, people affected by lung disease and people who use their breath in interesting ways (eg, free divers). It is an interdisciplinary project, working to find new ways of answering questions about breathing and breathlessness and their relationship to illness and well-being.

We may have gained a multifaceted perspective on the phenomenon of breathing by adopting an interdisciplinary approach, but in what ways are we interdisciplinary, interactive as opposed to being simply additive—the descriptor attributed to multidisciplinary projects? Perhaps we have erred towards becoming more of a transdisciplinary project, defined as being multilayered, “transcending existing categories” by “showing how different epistemes might be productively inter-related”.3 The Life of Breath team have collaborated on conference panels, symposia and public engagement events, as well as coauthoring academic publications and reports. Here is the first time we have come together to describe an *interdisciplinary analysis* of a transcript of textual data. The data, representing the mismatch between how breath is measured on a respiratory questionnaire and how respiratory patients experience their breathlessness, are already transdisciplinary, using respiratory questionnaires to explore the dissonance between clinical expertise and expertise by experience,4 exploring ideas of “testimonial injustice” in the clinic.5 The interdisciplinary analysis that we propose is underexplored because there is an explicit combination of analytical tools from paradigms where epistemological assumptions vary.6 For example, whereas a literary scholar reads the textual data as a language object, an anthropological scholar reads the textual data as a co-created encounter of meaning-making. Indeed, a recent survey of interdisciplinary research found it more evident across different *scientific* disciplines while collaboration across “distant disciplinary divisions” is much more rare.7

Discussions about methodological pluralism often imply that methodological rigour depends on the maintenance of boundaries between disciplines and their methods. The permeability of those boundaries is seen to either threaten rigour or gloss over methodological difference, especially when some argue for the “mixing at the level of data” and also “mixing of the realist, interpretative and constructionist paradigms”.8 In contrast to critiques of this “bricolage”9 is the view that that resistance to methodological pluralism runs the risk of methodological fetishism.10 To counteract such fetishism, others advocate methodological emancipation, involving de-coupling qualitative methodologies from their roots in social science and embedding them instead in the disciplinary worldview of applied health research.11 Some go further still, arguing that methodological boundary construction should be conceived as immutable, involving an osmotic process in which

“elements of particular analytics move in and through one another […] when boundaries are conceived as mobile and those between methods are understood as determined by the object of knowledge”.12

In the Life of Breath project, our object of knowledge is the lived experience and perception of breathlessness. We draw on multiple disciplines to reveal how breath and breathlessness are portrayed, for example, in literature or historically in the clinic in order to make the experience of breathlessness less invisible to the public and clinical gaze.

This article explores how five disciplinary contexts brought together in the Life of Breath project respond differently to the same data. The culture of our discipline is the binoculars through which we ‘see’ the data we encounter. We were curious to see if the condition and context of an interdisciplinary study had influenced the tint of our analytic gaze and the extent, if any, of transmethodological osmosis.13

## Research methods

The qualitative interview data for this article were collected by AM as part of the Life of Breath study. The interviewee was recruited from a Breathe Easy group and had been diagnosed with chronic obstructive pulmonary disease (COPD) 5 years previously. The study used cognitive interviewing techniques14 to explore patterns in answer mapping and comprehension of a recently developed scale for measuring breathlessness, the Multi-dimensional Dyspnoea Scale (MDP).15 One transcript was selected for the interdisciplinary analysis after an excerpt from it sparked a lot of discussion across the research team at a meeting in 2016 (at which all the coauthors for this article were present). The transcript is part of a bigger data set which has been analysed thematically by AM for another article.16 The selected transcript resonated thematically with a subset of other transcripts in the larger data sample. It is feasible that the findings from the analysis presented here could subsequently be developed into a transdisciplinary thematic framework and applied in the analysis of other interview transcripts in the data set by one researcher. This could be one method to adopt for future projects wishing to use transdisciplinary approaches to analysing data. For this project, the selected transcript was transcribed, anonymised and sent by email to all the coauthors. Using a short excerpt from one interview transcript allowed us to explore the interviewee’s account from the perspective of multiple disciplines while creating both continuity for the reader of the finished article, as well as opportunities to more easily compare how disciplinary responses overlapped or not. This approach has been successfully executed recently in this journal.17

## Patient and public involvement

The development of the research question for the larger cognitive interview study from which the data explored in this article were taken was developed alongside patients living with COPD. Patient and public involvement (PPI) experience suggested that clinicians often fail to enquire about the symptom of breathlessness in primary care settings and that the experience of completing questionnaires in clinical settings does little to meet concerns and needs about breathlessness. Meetings with adults living with respiratory illness were consulted through regional Breathe Easy groups and public engagement activity. The findings from this article will be made available in a shortened format to chairs of the Breathe Easy groups who were involved in the PPI consultation process.

## Transcript analysis

Each coauthor responded to the textual data excerpt, drawing on their disciplinary context and its methodological approach. Coauthors, as analysts, were given very little instruction except to write a reflective response to the data, including discussion on how their disciplinary context greets the subject of breathlessness and whether this has been influenced in any way by the interdisciplinary context.

## The interviewee: a breathless gaze

The interviewee was a man in his mid-seventies who for the last 5 years had been experiencing breathlessness. The interviewee had a diagnosis of a chronic respiratory condition. He had started smoking at the age of 26, had stopped in his sixties but taken it up again in the year before the interview because he felt hopeless. At the time of interview, he was more reflective though things were still obviously difficult for him. He was only able to walk eight metres before becoming painfully breathless. He used a mobility scooter to accompany his wife shopping and could not leave the house without her support, apart from going to the post box and back a few metres from his home. He was well supported by loving friends, part of a local Breathe Easy group, when he was well enough to attend. We re-produce here (box 1) an excerpt from a verbatim transcription of him thinking aloud his thoughts as he ponders how to respond to two questionnaire items on the MDP: “my breathing requires muscle work or effort” and “my breathing requires mental effort or concentration”. As he thinks aloud, he repeats the phrases of the questionnaire items many times.

Box 1 **The interviewee: data excerpt**

‘You said I was normal looking, quite fit for a 76 year old. And then to walk eight metres, come back, he’s gone… When you breathe, do you make a conscious effort of trying to breathe? No. I don’t make a conscious effort of trying to breathe, except when I walk that eight metres, then I’m gasping for breath. And (takes a deep breath) I’m not making a conscious effort to do it. Even after I’ve walked that eight metres. I’m sat here now, I’m, I’m shaking. I’m just trying to breathe normally to get my breath back so as (deep breath) I’m acting normally. (“My breathing requires muscle work or effort”?) I don’t think it, I don’t think it does. I, I think, if you were—if that was a broken leg and you were trying to get that broken leg back quicker or better, you’d exercise it…

It’s the same with your breathing. You can’t exercise your brain—or how can you? You can do a puzzle or something like that to keep it in working order. But if you—if I stop breathing now, I’m dead. And there is no conscious effort. It’s, it’s, er, my brain is controlling my breathing, making me breathe. I’m not making a, er, a conscious effort of using muscle to work or, or effort. I suppose I am…

Right ‘when exercising, my breathing requires muscle effort or work’, I don’t think it does, it just (mimics) ‘gasps, gasps’, is that me? My muscles working or my brain telling my lungs to start working, but that’s not muscle effort, well I don’t think so anyway (sighs), to me my lungs haven’t got any muscles, have they? Your diaphragm, if you breathe in, deeply from your tummy up, that pushes your lungs up and empties your lungs and then back down again, but that’s your diaphragm working, not your lungs working isn't it… My breathing requires intense (clears throat)—I’m not getting nothing, my chest feels constricted, my, er—I am breathing a lot, yeah, I could say 10 (on the questionnaire) to every one of them (items) if I’m exercising. But if I’m sat here now, it doesn’t take any effort to breathe. So I, I could do two sheets of that, one when I’m exercising and one when I’m not. No, I mean if I put resting there, (coughs) reading, I could put no effort at all, it, it just happens. Um, I mean a little child when he’s freshly born, he doesn’t make any specific effort to breathe. I mean it’s a natural thing to breathe. I mean.

I don’t think breathing does require muscle effort, except, if you’re breathless, and if you are exercising, you are trying to fill your lungs up and calm down, you’ve seen what i’m like after exercising, and you (breathes deeply) then you’re trying to get, fill your lungs and calm down. I mean you’ve seen what I’m like after exercising, you come back in again and you go (breathes deeply) and you’re gasping for breath, you take deep breaths, as much as you can until it gets painful, to try and breathe…‘my breathing requires mental effort’, no it doesn’t, it just happens, my brain is telling me that, my lungs aren’t telling me to breathe”.

## Findings

### Anthropological response

“My breathing requires mental effort or concentration”, “My breathing requires muscle work or effort”. Why are these questionnaire items problematic? Is it because both raise questions on the boundaries between a sense of ‘me’, ‘my breath and/or body’ and a conscious ‘I’? Or what Analayo, writing from the meditative tradition, describes as the conditional interrelationship between physical and mental phenomena: “as breathing is a process that can take place either involuntarily or deliberately, it stands in a distinctive conditional position in regard to body and mind, and therefore *offers an opportunity to contemplate the conditional interrelationship between physical and mental phenomena*”.18

For this man living with COPD, the questionnaire items on muscle effort or mental effort trigger a reflective discussion on selfhood and the breath as he explores “who is breathing and who am I when I am struggling to breathe”. Pathology and breathlessness, like meditation, appears to offer its own doorway into contemplating the interrelationship between physical and mental phenomena.

He begins by noticing that as soon as he becomes breathless, the “normal” self he identifies with is absent: “he’s gone”. These two items are problematic in part because his respiratory limitation is not ‘who he is’ all the time. His pulmonary disease disrupts his biographical flow several times a day.19 He is fine when at rest, reading a book, but could not walk eight metres without getting extremely breathless. What we see unravelling in this extract is *a contemplation of his two breath selves*. Each breath self could complete its own respiratory questionnaire because experientially they are two separate persons who appear unreconcilable. There is resonance here with work on bodily doubt that distinguishes the state of bodily certainty, in which the possibility of actions is taken for granted, from bodily doubt, where the certainty is replaced by a sense of uncertainty about whether the body can be trusted to perform as before.20

Further on in the account, the interviewee mimics gasping breaths and asks “is that me? My muscles working or my brain telling my lungs to start working?” He separates out his sensory experience (gasping) from a sense of person (me), from his physical body (my muscles) and all three from his command body (my brain). We see in his statement “my brain is making *me* breathe” and “my lungs aren’t telling *me* to breathe” that there is an expressed sense of ‘I’ (selfhood) that is separate from his bodily organs (lungs and brain) and bodily processes (breathing). The organs are given agency, they can force action, they can ‘make’ and ‘tell’ or ‘not tell’ but underneath this experience is a perception of a fixed ‘me’. Why does this matter?

It matters because according to the logic of early Buddhist thought, what causes suffering is our attachment to the idea of a fixed self and a reification of perceptual and sensory experience as ‘mine’. This is not dissimilar to general tenets of therapeutic process which involves being able to stand back from the part of us that causes our suffering and see it differently. It “implies the conceptual division of the self” in which the part of us which causes our suffering, in this case breath or lungs, are “given a personality with [their] own needs and will.”21 Crucially, suffering is eased when there is a reconciliation of the divided self, “a reintegration of the self’s two parts […] a self which needs to become conscious of its internal conflicts to bring them into relationship and eventual harmony”.22 Elsewhere, we have explored how arts and health approaches may facilitate the journey from ‘breath as antagonist’ to reconciliation.23 Responding to even a short excerpt of textual data demonstrates that breathlessness is more than just a vital symptom, it opens up questions on ‘what makes a person’. As Faull and colleagues suggest, breathlessness needs to be treated as something more than pathophysiology: “the point is to view breathlessness as something that might not directly correspond to airway pathophysiology and which may need to be treated both in parallel and independently of the lungs”.24

### Philosophy response

From a philosophical–phenomenological perspective, illness changes the lived experience of the ill person. It disrupts routines and habits and draws attention to bodily phenomena that are otherwise tacit.25 The body’s transparency, to use Sartre’s term, is replaced by an opaque, troubling presence. The body can no longer be taken for granted and bodily functions require attention, thus occluding the previous transparency. Within this framework, the interview excerpt reads like a lament for that lost transparency, accompanied by a sense of confusion, perhaps disorientation, as the interviewee tries to make sense of his disrupted embodiment. In this commentary, I’d like to point to the series of rifts that characterise breathlessness, which I have also analysed recently elsewhere.26

The first is the gap pointed out by the interviewee between normal (resting) breathing and pathological (on exertion) breathing. The interviewee states “I could do two sheets [questionnaires] of that, one when I’m exercising and one when I’m not”. This expresses the duality of his breathing experience: when resting, it is restful, easy, automatic, effortless, but becomes restless, stressful, difficult, explicit, on exertion. While normal breathlessness feels controlled, willed and often pleasurable, pathological breathlessness is felt as loss of control, frightening and distressing. I believe that pathological bodily experiences are not simply extreme forms of normal bodily experience but are underpinned by different qualitative and interpretative elements. Healthy breathlessness is temporary, normal and expected. Pathological breathlessness is chronic, abnormal (disproportionate to the task, eg, walking eight metres) and unexpected. It is out of the ordinary. I think that is what the interviewee means when he speaks of ‘two sheets’.

Another rift is revealed in the conflicting attitudes and emotions characterising the interviewee’s relationship to his breath. He is unsure whether muscles are involved in breathing; he is uncertain as to whether one can exercise one’s brain; he finds the difference between breathing at rest and on exertion baffling. The text is full of gaps, gasps, abrupt halts, question marks: ‘is that me?’ […] ‘my lungs haven’t got any muscles, have they?’ […] ‘I don’t think it, I don’t think it does’. It reads a little like a Beckettian monologue: full of about-faces, abrupt stops, gaps, unfinished sentences. The text itself is breathless. The interview performs the breathlessness of the interviewee. The interviewee tries to describe the experience of breathlessness, but language seems to fall short of the task. “… and you, then you’re trying to get, fill your lungs and calm down. […] you come back in again and you go… and you’re gasping for breath, you take deep breaths, as much as you can until it gets painful, to try and breathe…” The text is textured by sounds of breath: deep inhalations, throat clearing, gasping and sighing. These are all breath sounds, voiced exhalations. And they punctuate the text excessively, mirroring the continued yet failed attempt to tame, calm and understand the breath.

The final rift is that of failure(s). The rift between attempting to understand breathlessness and failing to grasp it. And between attempting to control the breath and succumbing to breathlessness. Given this internal turmoil, it is not surprising that breathlessness remains poorly understood by health professionals and those who do not suffer from it. This excerpt exemplifies how difficult it is to speak about breathlessness and how difficult it is to speak when breathless. The two difficulties intertwine in profound and complex ways, making breathlessness invisible. What the interview enacts is the difficulty of expressing this intimate yet overwhelming experience. It is hard to put into words and it is also hard to get the words out when one is so short of breath.

### Literary response

References to breath, when they appear in literary texts, bear an uncanny resemblance to markedness.27 Markedness is a contested linguistic category that differentiates two forms of an utterance: its dominant, assumed form (unmarked), and its variant, whose deviation from the dominant marks it as different. When Nikolai Trubetzkoy developed markedness theory, in his groundbreaking work on phonology, he identified unmarked, or dominant, terms as those utterances whose sound features deviated least from normal breathing.28 In literary texts, characters are assumed to breathe. Any reference to breath is therefore marked: it plays some role, significant or not, in the development of a narrative, character or plot. A parallel concern with markedness emerges in the interview. “See”, he says, “you could write a book, like that, on how you’re feeling and everything else, but you couldn’t put it down on one simple form”.

The text challenges the interviewee’s continued, uninterrupted sense of identity as a sufferer of breathlessness by using different pronouns to refer to himself. He begins the passage by referring to himself as “I”, a person who looks fit for a 76-year-old, only to shift that identification to “he”: this person, “he”, is “gone”. At several moments, the interviewee will invoke himself in the first person, but go on to describe his experiences of breathlessness as the conditions of a second person “you”. If these moments of dissociation help the interviewee to address deeply affecting conditions in a clear and honest fashion by taking them at some remove, they are also stylistically significant since they also perform precisely that alienation from the self that seems to happen after the interviewee walks eight metres: “he’s gone”.

Corresponding to these moments of stylistic transfers in identification are those moments when the interviewee reflects on the apparent autonomy of his organs. The interviewee describes his brain as “telling” him to breathe, as if this most central of organs were not fully incorporated into his self. This site of control is contrasted with the lungs, which “aren’t telling me to breathe”. These are linguistic parallelisms: semantically and/or syntactically similar phrases whose similarities highlight conceptual continuity or discontinuity. In this case, different body parts are paralleled through an instance of personification. In general, personification is the ascription of human qualities to nonhuman entities; here, it is the ability to speak. In this case, the parallel emphasises the interviewee’s understanding that the locus of control for breathlessness is the brain.

However, on closer analysis of this parallel against other references to control, the emphasis shifts from determining the locus of control to rendering it indeterminate. When imagining connexions between breath and the body, the locus of control is difficult to identify. While “my brain is controlling my breathing, making me breathe”, he reflects that “I’m not making a, er, a conscious effort of using muscle to work or, or effort”. He immediately contradicts this assertion—“I suppose I am…”—precisely because “that’s your diaphragm working”, he muses, “not your lungs working, isn’t it”. If parallel phrases about control helps to track the way in which its locus shifts, the consequence is to repeat those identification transfers already mentioned: “is that me? my muscles working or my brain telling my lungs to start working, but that’s not muscle effort, well I don’t think so anyway (sighs), to me my lungs haven’t got any muscles, have they?” The interviewee’s repeated reference to “muscle effort or work” frames a problem that exceeds its terms. “Doing work” may be repeated through the passage, but it fails to describe, consistently, the experience of breathlessness, an experience that seems to unmoor subjectivity and agency in language as much as in life.

### Clinical response

The process of completing the questionnaire appears to prompt the interviewee to reflect more closely on their control of breathing. Intuitively, this feels like a good thing. However, this can lead to overinterpretation of the questions. Clinical questionnaires often prompt the person completing them not to do this and to go with the most instinctive answer. In general practice, breathlessness questionnaires are not used to assess the patient. This potentially gives the patient more opportunity to express their experience of breathlessness in their own words. However, in the absence of routine questions about breathlessness, it means that GPs rarely ask about this experience and end up with even less information about the impact of breathlessness on the patient’s life than that (imperfectly) captured in a respiratory questionnaire. In this interview, the patient expresses profound, repetitious loss. He has lost the “normal” man he was before his COPD severely limited his ability to walk more than a few metres without the onset of breathlessness. The interviewee expresses frustration with the questionnaire “I could say 10 to every one of them if I were exercising…”, “I could do two sheets of that, one when I’m exercising and one when I’m not”. It is almost impossible for a questionnaire to capture breathlessness in every situation. He goes on to articulate clearly the limitations of questionnaires in capturing lived experience: “You could write a book, like that, on how you’re feeling and everything else, but you couldn’t put it down on one simple form”. Clinicians, particularly specialists, can be justifiably criticised for being too focused on the physical, ignoring the psychosocial and by interpreting symptoms through their particular clinical interest (heart, lungs). However, patients often bring similar prejudices into the clinic. Demonstrated, we think, in this case by assuming that all the experience of breathlessness relates exclusively to the lung, ignoring the role of the muscles of respiration. Many passages in the transcript focus entirely on the lung, often detailing the anatomy, disconnecting this from any other part of the body.

In critiquing the question about mental effort or concentration being required for breathing, the interviewee recognises the subconscious automatic control of breathing, but dismisses the role of conscious control, not sharing the experience of some patients with chronic breathlessness who speak of having to make a conscious effort to take deeper breaths. Clinically, we know that there is both subconscious automatic control of respiration, taking place at the level of the brain stem, driven by physiological feedback from the lung and cardiovascular systems. The conscious control of breathing takes place in the higher cortical centres, often sharing pathways related to emotion and the processing of pain. it is interesting to observe that the interviewee conflates muscle effort with conscious control of breathing: “My breathing requires mental effort or concentration, no, it doesn’t. It just happens. My brain is telling me that. My lungs aren’t telling me to breathe”.

“Um, you take deep breaths—well, as much as you can until it gets painful”. We often neglect to actively enquire about pain when we meet people in clinic with lung disease. Perhaps incorrectly leaving this exclusively to the cardiologists pursuing the diagnosis of ischaemic heart disease or heart attacks. However, there is empirical evidence that people living with advanced COPD report chest pain as one of the most frequent symptoms. In general practice, we do ask about pain in relation to breathlessness, but exclusively to consider the possibility of angina. Reading the transcript was a reminder to me that I do not enquire about pain in the context of chronic breathlessness.

The invisibility of breathlessness at rest is a theme emerging from this interview. It reminds me of the times that I have not seen how burdensome chronic breathlessness is when the patient is sitting comfortably in front of me. I think the interviewee might be telling the interviewer about the invisibility of breathlessness, particularly at rest: “I mean you’ve seen what I’m like after exercising: you come back in again and you go (breathes deeply) and you’re gasping for breath”.

### The body historical response

The participant experiences his most “natural” and effortless breathing to be like that of a baby, entirely lacking in any kind of painful intention or reflexivity: “I mean a little child when he’s freshly born, he doesn’t make any specific effort to breathe. I mean it’s a natural thing to breathe”. The participant seems to feel that all is well when breathing happens of its own accord, “naturally”, under control of the “brain”, and not the “muscles” (which he associates with volitional breathing or breathing of which he is painfully aware). Both current physiological models and forms of common sense, as expressed by the breathless interviewee, take breathing to be fundamentally involuntary, governed by the central pattern generator of the brainstem. But this way of experiencing the breath has a history. And that history reveals something about how much freedom is imagined to be possible for the human body and, therefore, for a human being.

Much like the interviewee, the Roman physician and philosopher Galen thought of the muscles as “the instruments of the will”, an imagination of the physiology of human autonomy with profound implications for many other aspects of body and self.29 Kuriyama argues that making the muscles the embodied source of the human will was tightly linked with anatomical thinking itself; the idea of the body as a collection of tools or “organs”. Galen recognised that breathing could manifest “automatically”, but he also thought of volitional breathing as deeply rooted in the human organism; an apocryphal story he first told would reappear in various forms well into the eighteenth century: slaves, lacking any other tool of protest, foiled their masters’ plans by holding their breath until they died.30 Breathing was taken to be such a volitional thing that it gave any breather ultimate determination over their own life or death.

But by the late nineteenth century, long unquestioned modes of human uniqueness including the faculty of “will” were under threat from new forms of materialism, and mechanistic and reductionistic models of body and self, including certain interpretations of Darwinism.31 When it came to human breathing, in the 1889 edition of Charles Darwin’s *The Expressions of the Emotions in Man and Animals*, a work in which he approached apparent human emotional and expressive uniqueness from an evolutionary perspective, he wrote that breathing was fundamentally, and ideally, *not* wilful: “Respiration is partly voluntary, but mainly reflex, and is performed in the most natural and best manner without the interference of the will”.32 Breathing was “mainly reflex”, much as the interviewee described.

**Figure 1:**
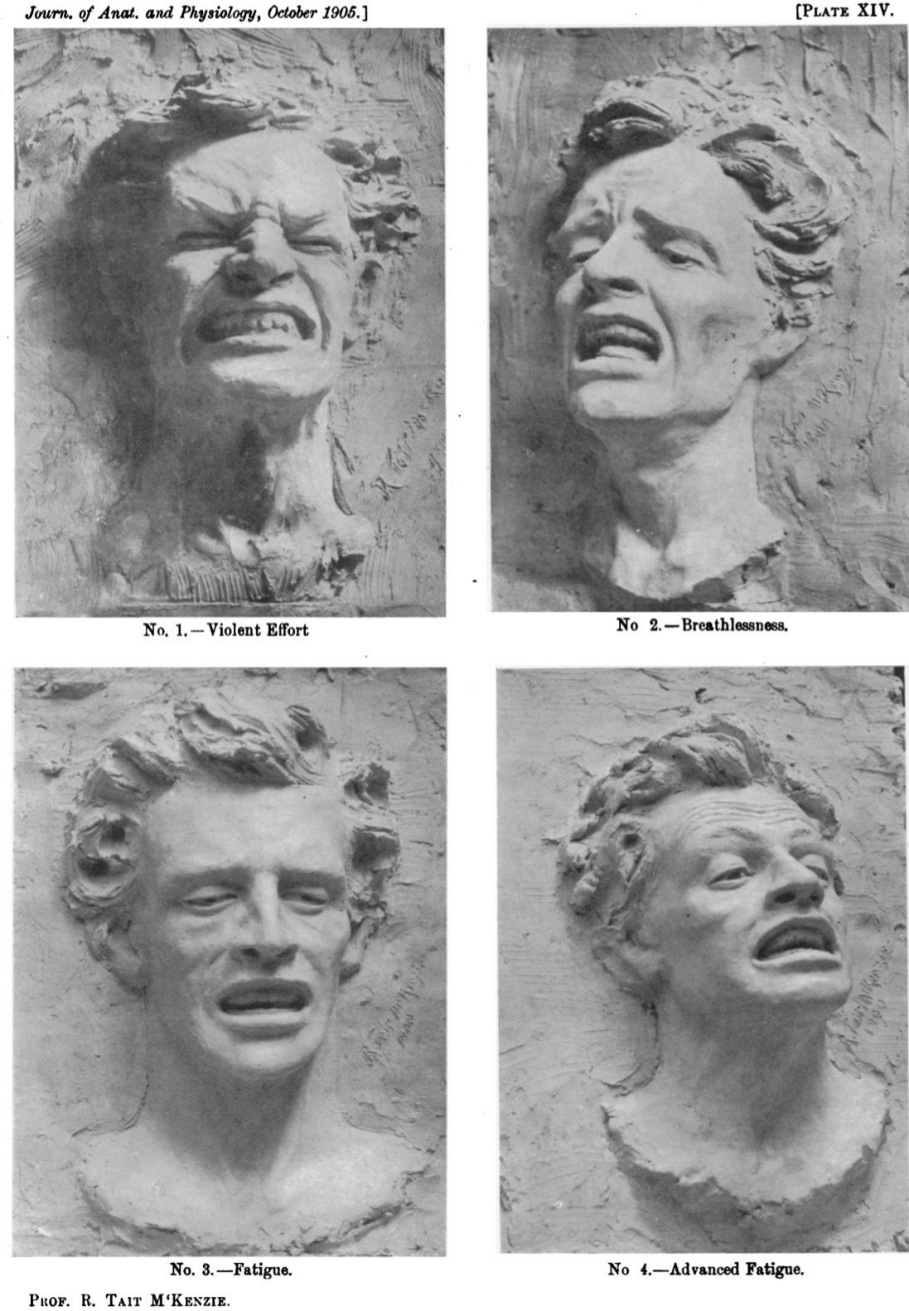
The Facial Expression of Violent Effort, Breathlessness, and Fatigue53

Having closely studied both Charles Darwin and Charles Bell’s anatomies of expression,33 in 1905 R. Tait McKenzie, a Canadian professor of the new discipline of physical education, and, as it happened, an amateur sculptor, created a series that illustrated the stages of the peculiar fatigue of breathlessness; it was, he wrote, a composite of Charles Bell’s work on the “anxiety associated with bodily distress” together with the addition of “gaping mouth and expanded nostrils”.34, 35 He anatomised breathlessness in the following way:

“In this mask (no. 2 in figure 1) we have the typical face of the breathless man. The smoothness of the forehead is broken by wrinkles spreading out from the inner end of the updrawn eyebrows, where the general direction is just the reverse of that seen in violent effort; they are drawn upward and inward by the corrugator supercilii, the muscle of pain, which always acts in grief, mental distress, anxiety, and bodily pain”. (p.53)36

It is interesting that for the earliest of McKenzie’s sources, the early-nineteenth-century anatomist Charles Bell, the corrugator supercilii muscle was “a muscle peculiar to human expression”.37 Though Darwin would later argue against this view, for Bell human facial expression was a matter of human uniqueness, and one of the key muscles at play in the peculiarity of human pain was a central feature of the pain of breathlessness, the corrugator supercilii: “The most remarkable muscle of the human face […] it knits the eyebrows with an energetic effect, which unaccountably, but irresistibly, conveys the idea of mind”.38 Before breathing became a reflex, or perhaps, in the case of breathlessness, a reflex gone wrong or pushed beyond its reasonable limit, the pain of breathlessness wasn’t just any pain; it was a kind of pain with a particular human element. It conveyed “the idea of a mind”.

Our imaginations of the body matter. Most poignantly, as we see behind the account of the interviewee’s breathlessness, they instruct us in how to suffer and determine the nature and meaning of that suffering. History reveals that these models change across time and place, and yet they are so intimate as to be hardly distinguishable from our very selves. But there is indeed a tiny gap, a niggling suspicion: “‘gasps gasps’, is that me?… my lungs haven’t got any muscles, have they?” To look historically at the body is to look into that space of possibility. When we consider the multiplicities of human breathing, we are at once considering the possibilities for human autonomy: which of many possible views gives us the most freedom to face the inevitability of breathlessness?

## From methodological pluralism to multidisciplinary gaze

We have chosen to frame the responses to the data in terms of *disciplinary gaze* as opposed to *methodological approach* because each of the researchers responding to the data have an epistemological position partly determined by their disciplinary training.39 The work reported in this article took place in the fourth year of the Life of Breath project. It is likely that by this point in time a greater understanding of each other’s disciplinary gaze had already occurred.

### The anthropologist’s gaze

*AM:* The anthropological discipline shapes my approach to analysis through its prioritisation of three things: the thick description of the lived experience, the interpretative nature of analysis and the importance of the encounter of the researcher and the participant in the production of knowledge and ‘data’. Prior to joining the Life of Breath project, my work had explored mindfulness based approaches to supporting those living with breathlessness. Mindfulness comes from a Buddhist epistemology in which the breath is used as an object of meditation because it is seen as a doorway into self-observation. As a direct encounter with the ideas and work of OW through the Life of Breath project, I became interested in how breathlessness (as opposed to a meditative breath) may also lead someone to explore personhood. I approached the data with ideas about how we breathe life into personhood and with these ideas in mind, subsequent encounters with the data inspired an enquiry into how we differentiate (if we do at all) between ‘me, my breath and I’.

### The philosopher’s gaze

*HC*: Philosophers are interested in concepts and ideas and how these shape our understanding of the world. The approach offered here, a phenomenological approach, has much in common with qualitative research. It aims to describe, not explain. It views the person as part of a complex web, and hence as situated within a world and engaged with other people. First and foremost, it views the person as embodied and her terms of embodiment as the foundation and structure of her experience. My textual analysis is an example of a philosophical approach to a text. I was looking for key words and ideas that could then be abstracted into a more general theme. In this case, the theme of ‘rifts’ emerged from my reading of the text.

The interdisciplinary research practised by the Life of Breath team has been profoundly significant for me. We set up the project with the belief that if we want to shed light on a phenomenon such as breathing, that can only be done using a network of disciplines and approaches. Being able to learn from colleagues from other disciplines gave me a critical perspective on my own discipline, as well as being deeply edifying in ways that are very different to engaging with others within my own discipline.

*TW*: As a philosopher, approaching breathing and breathlessness from the philosophical tradition prepared me to delve into historical accounts of the breath as *pneuma* in the pre-Socratics, for example, through to presentations in contemporary philosophy. However, the paucity of such explorations in Western philosophy revealed that not only did the breath need to be re-covered and drawn forth, but that the work uncovered within this multidisciplinary group were vital for this. I found myself holding my breath when reading the data excerpt, echoing Levinas’s reflection that the lungs may be the truly ethical organ: it is through the breath in the encounter with the Other that we can be summoned to put our own agency and interests behind those of the Other.40 And what could be more ethically demanding than watching another gasp for breath? When someone is in such distress, struggling for breath, that demand is certainly undeniable.

### The literary scholar’s gaze

*AR*: My response is conditioned by my training as a literary scholar who focuses on rhetoric, style and critical theory. I read the text first and foremost as a language object, conditioned by narrative coherence, linguistic parallelisms and metaphor. I encounter the interviewee primarily as a character, whose materiality and weight derives from descriptive language, mediating this abstraction by recalling that the interviewee is a real person describing real experiences. This mediation allows me to temper a first-order formalism, treating the passage as text, with a second-order ethical imperative, to remember the person behind the text. This play between the formal and the ethical is complicated by two further concerns: identification and context. I am trained to reflect on the ways in which readers identify with characters. In relation to the transcript, I am interested in the textual cues that provoke us to empathise with the ‘character’, and, by extension, the interviewee, as part of a language world. I am also trained to respond to language as context specific. Since I generally work with language objects that are crafted over time, my critical tools are ill-adapted to analysis of an interview transcript since I grant the precise meaning of the words a greater significance in developing context (narrative coherence), linking ideas (linguistic parallelisms) and figurative imagining (metaphor) than might be intended in an interviewee’s spontaneous reactions to interview questions, no matter how long they might take to consider this response. Working with the LoB team, where the patient voice is granted a centrality, has pushed me to reflect more on the pragmatics of everyday usage of breath language than is commonly suggested by literary scholarship on breath as a figural device.41

### The clinician’s gaze

*JD*: As a specialist respiratory physician working in a busy teaching hospital, I supervise the care of acutely unwell patients admitted to hospital with lung disease and meet people in outpatient clinic sent to me by their GP and other healthcare professionals for assessment and advice about lung disease and breathing problems. While medical school and specialist training are grounded in traditional sciences such as physiology and biology, a good clinician appreciates that much of what we do is to synthesise subjective and ‘objective’ evidence accumulated through listening to our patients. We supplement this through physical examination and investigations including imaging and physiological measurements. It is the synthesis of this information, applied in the psychosocial context of the person sitting in front of us, that has long been considered the ‘art’ of medicine. Much of what is asked of me requires pragmatic output, in the form of diagnosis, advice on further investigations, monitoring and treatment. My clinical gaze in reviewing this transcript recognises the tension between the clinicians who develop these questionnaires, in an attempt to quantify breathlessness in a clinically meaningful way and the almost impossible task of capturing the truth of an individual’s experience of breathlessness at any given moment.

*GF*: As a GP working in a community-based practice, I am involved in an initial assessment of new-onset breathlessness, often making a diagnosis and starting treatment. I also encounter patients living with chronic breathlessness from lung disease (particularly COPD) or heart failure, as well as patients whose breathlessness is not based on any structural or physiological pathology. The general practice gaze, developed in postgraduate training, is explicitly biopsychosocial and this is applied to patients with new-onset or chronic breathlessness. This means that when patients present with a problem, my clinical gaze moves back-and-forth between their symptoms and signs, their emotional state, what I know or find out about their past experiences and their current living situation. The biopsychosocial gaze that I try to apply in my clinical practice has helped open me to the diverse perspectives on breathlessness shared and elaborated in the Life of Breath collaboration. Despite my attempting a biopsychosocial understanding of my patients’ condition, the languages of the medical body, human emotion and social context are not integrated—I switch between them in a consultation. Through my involvement in the Life of Breath project, I have become more aware of the epistemological and ontological leaps that this involves, the contingency of many breath-related diagnoses and measurement, and the potential for addressing breathlessness directly through enhanced understanding of biopsychosocial interactions.

### The body historian’s gaze

*OW*: There are good reasons for keeping clinical practice and the writing of its history separate. One compelling reason for boundary keeping is that historical thinking can be powerfully destabilising. Histories of medicine do not reassure practitioners that they stand on solid ground or that the trajectory of history leads from a benighted past into a knowing present. Is there a way to use interdisciplinarity, including history, to make clinical practice even better—more effective, more humane— than it already is?

As an historian of medicine and the body, my approach to this problem is to consider the many different bodies that are found in historical sources. To set these many modes of experience side by side, following Polish-Israeli biologist, physician and philosopher Ludwik Fleck42 in what he called *comparative epistemology*, allows for a recognition of the ways that our own experience—usually taken to be the simple fact of the matter—is precisely situated. Taking seriously both the nature and existence of different modes of being a body affords us a form of what David Bohm has called “proprioception of thought”, bringing contingent givens out of the shadows and into awareness.43 We don’t take “other” models and experiences of the body seriously for antiquarian interest, for the construction of a cabinet of curiosities, or even because inclusivity is the right thing to do. We take the actual diversity of ways of being seriously—be they bodies and body models, institutions, clinical practices—because they are the logic from which all that seems *given* follows. Holding multiple perspectives need not entail a kind of groundless relativism; instead, comparison brings hidden limitations to light and points the way to new and unexpected possibilities for human flourishing.

## Discussion

We explore why research into breathlessness may particularly benefit from an interdisciplinary approach. We then conclude by discussing the wider implications of the findings for interdisciplinary research within health and medicine by referring to current debates in the literature about the functions, successes and challenges of collaborative research between the humanities and sciences.

## Why is the questionnaire item hard to answer?

Each disciplinary gaze responds to the difficulty the interviewee experiences when asked “my breathing requires muscle work and effort”. The literary gaze (AR), paying close attention to what is spoken, notices how often the interviewee repeats the wording of the questionnaire item, “Doing work”. AR notes that it fails to describe, consistently, his experience of breathlessness. The clinical gaze (JD) likewise notes the interviewee’s frustration and the limitations of the questionnaire to “capture breathlessness in every situation”. They reflect on the role of questionnaires in clinical practice and clinician–patient communication about breathlessness (and its regular absence within primary care). The clinical response ‘explains’ what is scientifically known, pointing out where the interviewee’s telling of his experience differs from what “we clinically know” (eg, the interviewee’s dismissal of conscious control of breathing governed by higher cortical centres). The body historical response describes how the interviewee’s way of experiencing the breath (and articulating that experience) reveals a certain history about how the breath and its conscious and unconscious control has been imagined in science. For the clinician, scientific truth claims cast shadows on the interviewee’s accounts, whereas for the body historian the ‘powerfully destabilising’ nature of shifting science-truth-claims means the interviewee’s contradictions and difficulties in being able to answer the question “my breathing requires muscle work and effort” are reconciled by a situated historical “logic from which all that seems *given* follows”.

HC, OW and AM note the contradictions in the interviewee’s response, but their disciplinary training encourages an exploration of what is being communicated underneath the interviewee’s dismissal (and confusion) of the questionnaire wording. For example, for AM (anthropological gaze), the difficulty answering the questionnaire item is because breathlessness (like breath) forces the interviewee to consider the conditional inter-relationship between physical and mental phenomena. His very sense of personhood is undermined by the experience of breathlessness as he explores ‘who is breathing and who am I when I am struggling to breathe’. The historical turn in ideas of breathing are, OW argues, changing stories about the freedom imagined to be possible for a human being, each denoting a different kind of body and self.

The clinical gaze compares lay and medical epistemologies using the phrase “clinically *we know*”. There is an expectation (from within medicine) that the person can identify the nature of the sensation of breathing in a particular physiological function. This ‘particularising physiological function’ clearly confused the interviewee, raising questions about the nature of the sensation of breathlessness, how it is generated and influenced, which need unpicking beyond a purely physiological and clinical explanation. One impact of an interdisciplinary effort may be to lay the problematic nature of the questionnaire at the feet of medicine, rather than at the feet of the ‘unknowing’ patient.

## An interdisciplinary approach to breathlessness

Being asked to respond from a disciplinary perspective should immediately make the interdisciplinarian pause. Perhaps the task is not disciplinary (how do scholars from different disciplines respond to the data excerpt differently) but to consider *what disciplines are called into play* to interpret and analyse a transcript of breathlessness. ‘Medical humanities’ does not describe a discipline but rather an approach that recognises that the biomedical approach to understanding health and ill-health is insufficient to reveal its complexity and individuality, and that we need to draw on wider resources of understanding, particularly those that give insights into human experience, located within diverse histories, cultures, ethnicities, geographies and political contexts.

The starting point for the Life of Breath project collaboration was the uneasy tension that exists between the personal and the clinical languages of breathlessness44 and the clear evidence of a distinction between measured and experienced symptoms of respiratory disease.45 Breathlessness has been recognised as a multidimensional construct involving the perception of sensations, thoughts, feelings and behaviours.46 Yet cognitive interviews, such as the data excerpt used in this article, show the narratives of patients do not often fit neatly into clinical questionnaire categories. The questionnaires ‘speak’ a different language of breathlessness. The interviewee struggles to describe his sensory experience—Is it conscious? Does it feel like work? None of these descriptors on the questionnaire seem to cover it for him. An interdisciplinary approach to breathlessness has two tasks. First, to understand how it is that clinical science has arrived at the descriptors that the respondent is offered in the questionnaire, and second, to bring interdisciplinary insights about experience into dialogue with clinical science to develop more effective and accurate ways of helping.

## Implications for science humanities

In their critical imagining of what medical humanities could look like, Fitzgerald and Callard (2016) ask the question, what if disease were not a bodily fact that needed finer interpretation, but a way of describing a relation between a body, a history and an environment?47 In contrast to other interdisciplinary projects who may come together to share experiential understandings of working practice, for the Life of Breath project, it is the object of study itself, breathlessness, which embodies multiple disciplines and constructs, providing a “shared frame of reference” for the multiple disciplines which make up the Life of Breath team to coalesce.48 From the perspective of ‘science humanities’, Willis (2017) is uneasy with the Life of Breath project’s utilitarian approach to knowledge-making, one that is aimed ultimately at improving the treatment and management of breathlessness. Willis views the interests of medical sciences as a type of “disciplinary restriction (which) can often undermine attempts at new methodologies which would offer a richer ecology of knowledge”.49 Were the objective of the Life of Breath project to merely inform greater popular health literacy (eg, to educate and promote medical terminologies),50 we may have been guilty of our work being in service to a clinical disciplinary restriction. However, our aim is more ambitious, to acknowledge variations in language and differences in cultures of breathlessness as well as the histories of testimonial injustice in lung health; and then with interdisciplinary insights from the medical humanities and health sciences increase societal awareness and visibility of what breathlessness means, the variability in how it is expressed and how better to provide effective care.51

For example, members of the Life of Breath project team recently published a letter to the editor of the *European Respiratory Journal* on renaming breathlessness as ‘Chronic breathlessness syndrome’, in response to a proposal put forward by Miriam Johnson and colleagues in the same issue.52 The authors advocate working towards raising the profile of breathlessness within the palliative clinical encounter so they develop a “truly consensual terminology”, involving “experts by experience” in order to legitimise the historical and cultural specific influences on perceptions and experiences of breathlessness and avoid alienating people living with breathlessness.52 If breathlessness is going to be renamed so it has a clearer profile within clinical practice, making it easier for patients to discuss their ongoing breathlessness or clinicians to detect undiagnosed breathlessness, its renaming needs to take into account breath’s multidisciplinary nature and lifehood.

## Conclusion

Describing the work of Life of Breath as being concerned with the ‘disrupted songlines of breathlessness’ is a helpful way to view the multicontextual experience of breathlessness as well as the necessity of an interdisciplinary approach. A songline is a song used within Australian Aboriginal culture as a way to navigate across the land. By repeating the words of the songline, which describe the location of landmarks, the land is traversed. As songlines span the lands of several different language groups, different parts of the song are said to be in those different languages. Importantly, for its adoption in relation to breathlessness, language is not a barrier because the melodic contour of the song describes the nature of the land over which the song passes. The rhythm is what is crucial to understanding the song and its ability to cross boundaries (of discipline and experience).

The interdisciplinarian is best equipped to walk inside (and alongside) the lands of breathlessness, translating across borderlands wherever possible as she moves. This is because an interdisciplinarian is identifiable by her movement, the willingness to *depart* from her discipline, to not be landlocked by her discipline’s vantage point. Instead of having shouted conversations across borderlands, she must travel the fields of knowledge making with an intradisciplinary effort. An effort that acknowledges the critiques of the spatial logic of integration and recognises that “things do not have inherently determinate boundaries and properties […] rather boundaries are instead things we produce” (p. 40/41)35 across which she must make repeated crossings.

## Notes

1. Charlotte Hardman (2000). *Other worlds: notions of self and emotion among the Lohorung Rai*. (Berg Pub Limited).

2. Nollaig A. Frost et al. (2011). “Collective findings, individual interpretations: an illustration of a pluralistic approach to qualitative data analysis,” *Qualitative Research in Psychology* 8, no. 1: 93

3. Martin Willis, Keir Waddington and James Castell (2017). “ScienceHumanities: theory, politics, practice,” *Journal of Literature and Science* 10, no. 2:3

4. Alice Malpass et al. (2016). “Usefulness of PHQ-9 in primary care to determine meaningful symptoms of low mood: a qualitative study,” *British Journal of General Practice* 66, no. 643

5. Havi Carel and Ian James Kidd (2014). “Epistemic injustice in healthcare: a philosophical analysis,” *Medicine, Healthcare and Philosophy* 17, no. 4

6. Nollaig A. Frost and Sevasti-Melissa Nolas (2011). “Exploring and expanding on pluralism in qualitative research in psychology,” 115–119.

7. Willis, Waddington and Castell (2017), “ScienceHumanities,” 12.

8. Nollaig A. Frost and Sevasti-Melissa Nolas (2011). “Exploring and expanding on pluralism,” 94.

9. Ibid

10. M. Johnson et al. (2001). “Arguments for ‘British Pluralism’in qualitative health research,” *Journal of Advanced Nursing* 33, no. 2: 243–249.

11. Sally Thorne (2011). “Toward methodological emancipation in applied health research,” *Qualitative Health Research* 21, no. 4: 443.

12. Lisa Lazard, Rose Capdevila and Anca Roberts (2011). “Methodological pluralism in theory and in practice: the case for Q in the community,” *Qualitative Research in Psychology* 8, no. 2: 148.

13. Ibid

14. Dariusz Galasiński and Olga Kozłowska (2010). “Questionnaires and lived experience: strategies of coping with the quantitative frame,” *Qualitative Inquiry* 16, no. 4: 271–284.

15. Robert B Banzett et al. (2015). Schwartzstein, Paula M. Meek, Richard H. Gracely, and Robert W. Lansing. “Multidimensional Dyspnea Profile: an instrument for clinical and laboratory research,” *European Respiratory Journal* 45, no. 6: 1681–1691

16. Alice Malpass et al. (2016). “The body says it’: the difficulty of measuring and communicating sensations of breathlessness,” (under review).

17. Deborah Padfield et al. (2018). “Images as catalysts for meaning-making in medical pain encounters: a multidisciplinary analysis,” *Medical humanities*: medhum-2017.

18. Bhikkhu Analayo (2004). *Sattipatthana: the direct path to realisation*. Windhorse Publications,:131

19. Simon Williams (2000). “Chronic illness as biographical disruption or biographical disruption as chronic illness? Reflections on a core concept,” *Sociology of Health & Illness* 22, no. 1: 40.

20. Havi Carel (2013). “Bodily doubt,” *Journal of Consciousness Studies* 20, no. 7–8: 178–197.

21. Margaretta Jolly (2011). “What I never wanted to tell you: therapeutic letter writing in cultural context,” *Journal of Medical Humanities* 32, no. 1: 48.

22. Ibid, 21.

23. Penny Elspeth and Alice Malpass (2018). “Dear Breath: using story structure to understand the value of letter writing for those living with breathlessness: a qualitative study,” *Arts and Health: International Journal of Research and Practice*.

24. Olivia K. Faull et al. (2018). “Chronic breathlessness: re-thinking the symptom,” *European Respiratory Journal* 51, no. 1: 1702238.

25. H. Carel (2016). *Illness: the cry of the flesh*. Routledge.

26. Havi Carel (2018). “Breathlessness: the rift between objective measurement and subjective experience,” *Lancet Respiratory Medicine*

27. Arthur Rose et al. (2018). *Reading breath in literature*

28. Nikolai Sergeevich Trubetzkoy (1969). *Principles of phonology*: 146.

29. Shigehisa Kuriyama (1999). *The expressiveness of the body and the divergence of Greek and Chinese medicine*.

30. V. J. DERBES et al. (1953). “Voluntary death by breath holding” *T* *he New England Journal of Medicine* 249: 698–699.

31. Roger Smith (2015). *Free will and the human sciences in Britain, 1870–* *1910*. Routledge, 2015. Nikolai Sergeevich Trubetzkoy (1969). *Principles of phonology* Berkeley: University of California Press.

32. Charles Darwin. *The expression of the emotions in man and animals*, Paul Ekman ed. second Edition.: 42

33. Sir Charles Bell (1877) *The anatomy and philosophy of expression: as connected with the fine arts*. London: George Bell and Sons, York Street, Covent Garden, seventh edition. 128.

34. Robert Tait McKenzie (1905). “The facial expression of violent effort, breathlessness, and fatigue.” *Journal of Anatomy and Physiology* 40, no. Pt 1: 51.

35. Robert Tait McKenzie (1915). *Exercise in education and medicine*. WB Saunders Company.

36. McKenzie (1905). “Facial expression of violent effort,” 53

37. Sir Charles Bell (1877) *Anatomy and philosophy of Expression,* 128.

38. Ibid, 128.

39. Stacy M. Carter and Miles Little (2007). “Justifying knowledge, justifying method, taking action: epistemologies, methodologies, and methods in qualitative research.” *Qualitative Health Research* 17, no. 10: 1316–1328.

40. Paul Ricoeur and Matthew Escobar (2004). “Otherwise: a reading of Emmanuel Levinas's Otherwise than being or beyond essence” *Yale French Studies* 104: 82–99.

41. Arthur Rose et al. (2018). Introduction. In: Rose A, Heine S, Tsentourou N, Saunders CJ, Garratt P. *Reading breath in literature*. London: Palgrave.

42. Ludwik Fleck (2012). *Genesis and development of a scientific fact*. University of Chicago Press. Nollaig A. Frost and Sevasti-Melissa Nolas (2011). “Exploring and expanding on pluralism in qualitative research in psychology,” 115–119.

43. David Bohm (2013). *On dialogue*. Routledge.

44. Rebecca Oxley and Jane Macnaughton (2016). “Inspiring change: humanities and social science insights into the experience and management of breathlessness,” *Current Opinion in Supportive and Palliative care* 10, no. 3: 256.

45. Megan Wainwright and Jane Macnaughton (2013). “Is a qualitative perspective missing from COPD guidelines?," *The Lancet. Respiratory Medicine* 1, no. 6: 441.

46. Robert B. Banzett et al. (2015). *Multidimensional Dyspnea Profile*.

47. Des Fitzgerald and Felicity Callard (2016). *Entangling the medical humanities,* Edinburgh University Press:44

48. Martin Willis et al. (2017), “Sciencehumanities,”15.

49. Ibid, 15.

50. Nishtha Singh et al. (2017). “Respiratory disease terminology: discordance between pulmonologists and patients,” *Lung India: official organ of Indian Chest Society* 34, no. 1: 9.

51. Rebecca Oxley and Jane Macnaughton (2016). “Inspiring change,” 257.

52. Jane Macnaughton et al. (2018). “Chronic breathlessness: re-thinking the symptom,” *European Respiratory Journal* 51, no. 1: 1702331.

53. Robert Tait McKenzie (1905). “Facial expression of violent effort,” 51
